# The application of electrical impedance tomography and surgical outcomes of thoracoscope-assisted surgical stabilization of rib fractures in severe chest trauma

**DOI:** 10.1038/s41598-024-60392-0

**Published:** 2024-04-27

**Authors:** Yi-Jie Wang, Yuan-Ming Tsai, Yen-Shou Kuo, Kuan-Hsun Lin, Ti-Hui Wu, Hsu-Kai Huang, Shih-Chun Lee, Tsai-Wang Huang, Hung Chang, Ying-Yi Chen

**Affiliations:** 1 Department of Surgery, Tri Service General Hospital, National Defense Medical Center, Taipei, Taiwan, R.O.C.; 2 Division of Thoracic Surgery, Tri-Service General Hospital, National Defense Medical Center, 325, Section 2, Cheng-Kung Road, Taipei, 114 Taiwan, R.O.C.; 3 Graduate Institute of Medical Science, National Defense Medical Center, Taipei, Taiwan, R.O.C.

**Keywords:** Thoracic trauma, Surgical stabilization of rib fractures, ISS score, Age, ICU stay, Electrical impedance tomography, Diseases, Medical research, Signs and symptoms

## Abstract

Serious blunt chest trauma usually induces hemothorax, pneumothorax, and rib fractures. More studies have claimed that early video-assisted thoracoscopic surgery with surgical stabilization of rib fractures (SSRF) results in a good prognosis in patients with major trauma. This study aimed to verify the outcomes in patients with chest trauma whether SSRF was performed. Consecutive patients who were treated in a medical center in Taiwan, for traumatic events between January 2015 and June 2020, were retrospectively reviewed. This study focused on patients with major trauma and thoracic injuries, and they were divided into groups based on whether they received SSRF. We used electrical impedance tomography (EIT) to evaluate the change of ventilation conditions. Different scores used for the evaluation of trauma severity were also compared in this study. Among the 8396 patients who were included, 1529 (18.21%) had major trauma with injury severity score > 16 and were admitted to the intensive care unit initially. A total of 596 patients with chest trauma were admitted, of whom 519 (87%) survived. Younger age and a lower trauma score (including injury severity scale, new injury severity score, trauma and injury severity score, and revised trauma score) account for better survival rates. Moreover, 74 patients received SSRF. They had a shorter intensive care unit (ICU) stay (5.24, *p* = 0.045) and better performance in electrical impedance tomography (23.46, *p* < 0.001). In patients with major thoracic injury, older age and higher injury survival scale account for higher mortality rate. Effective surgical stabilization of rib fractures shortened the ICU stay and helped achieve better performance in EIT. Thoracoscope-assisted rib fixation is suggested in severe trauma cases.

## Introduction

Chest trauma remains a serious problem as the number of high-speed vehicle ac-cidents increase. Thoracic trauma occurs in approximately 60% of patients with poly-trauma and has a mortality of 20–25%^1,2^. Chest trauma refers to any injury to the chest region, which includes the ribs, sternum, and organs located in the chest such as the heart, lungs, and esophagus. Some of the common symptoms of chest trauma include chest pain, difficulty breathing, coughing up blood and chest tenderness or swelling. In severe cases, chest trauma can result in life-threatening conditions such as tension pneumothorax, massive hemothorax, flail chest, or cardiac tamponade. Initially, studies believed that ≤ 10% of patients with blunt chest trauma require surgical treat-ment, and the remaining patients can be treated conservatively^3–5^, with some patients receiving simple treatments such as appropriate airway assessment, oxygen support, tube thoracostomy, volume resuscitation, and adequate pain control. However, over the last two decades, surgery has evolved into one that is performed routinely, early in the post-injury course, to mitigate primarily long term outcomes such as pain and enable return to work and participating in recreational activities^6^.

Surgical stabilization of rib fractures (SSRF) is now a common procedure in most high-volume trauma centers. The benefits of thoracoscope assisted SSRF may be divided broadly into those related to the repair of rib fractures and to adjuncts to rib fracture repair. With respect to the former, theoretical benefits include improved visualization of rib fractures (particularly in posterior and subscapular locations), minimization of trauma to overlying muscles and nerves, and minimization of trauma to intrathoracic structures. With respect to the latter, theoretical advantages include evacuation of retained hemothorax, guided placement of locoregional anesthesia and chest tubes, and identification and repair of associated intrathoracic injuries^7^. Besides, the EIT uses electric current to evaluate the distribution of alternating current conductivity within the thoracic cavity. It is a non-invasive, bedside radiation-free functional imaging modality for continuous monitoring of lung ventilation and perfusion. So, we used EIT for precise evaluation of pulmonary ventilation status before and after treatment. Thus, this study aimed to verify the outcomes of patients with severe chest trauma admitted in the ICU whether SSRF was performed.

## Materials and methods

### Patient selection and study design

We performed a retrospective data collection of all polytrauma patients with an injury severity scale (ISS) ≥ 16 and AIS ≥ 2 in more than one injured body region admitted to the emergency department of Tri-Service General Hospital, a medical center in Tai-wan, from January 2015 to June 2020. The inclusion criteria were blunt chest trauma with an AISthorax ≥ 2 and admitted to the intensive care unit initially. This study focused on the severe chest trauma cases, and the exclusion criteria were AISthorax < 3, AISlimb > 3, AISabdomen > 3, and AIShead > 3 and inadequate follow-up data. This study was approved by the Institutional Review Board/Ethics Committee of our hospital (1-108-05-165).

SSRF is a procedure that involves fixing the broken ribs with titanium plates and screws with RibFix Blu™ Thoracic Fixation System and MatrixRIB™ Fixation System in our patients. SSRF can improve the outcomes of patients with severe chest trauma by reducing pain, respiratory complications, and hospital stay. According to the current consensus of indications of SSRF are: (1) Flail chest is a traumatic condition of the thorax. It may occur when 3 or more ribs are broken in at least 2 places. The flail of chest cause paradoxical movement of chest wall. (2) Multiple rib fractures: Patients with more than three rib fractures are more likely to benefit from SSRF, especially if the fractures are displaced or flail. (3) Severe pulmonary contusion: Patients with lung injury caused by blunt chest trauma may have impaired gas exchange and ventilation. SSRF can help restore chest wall stability and prevent further lung damage. (4) Hemothorax or pneumothorax: Patients with blood or air accumulation in the pleural space may require thoracoscopic drainage and evacuation. SSRF can be performed at the same time to fix the rib fractures and prevent recurrence. (5) Nonunion or malunion: Patients with delayed healing or abnormal healing of the rib fractures may suffer from chronic pain and deformity. SSRF can correct the alignment and promote bone healing.

### Parameters

The survival rate was compared according to age, sex, involvement or nonin-volvement of a trauma team, and whether SSRF was done. We use different scales including the injury severity scale [ISS], new injury severity score [NISS], trauma and injury severity score [TRISS], and revised trauma score [RTS] to evaluate these patients with severe blunt trauma and distinguished which one may be more appropriate and relevant to the survival rate. The ISS is an anatomical scoring system that provides an overall score for patients with multiple injuries^8^. Each injury is assigned an abbreviated injury scale (AIS) score and is allocated to one of six body regions^8^. The RTS is made up of three categories: Glasgow Coma Scale, systolic blood pressure, and respiratory rate^8^. The NISS is simply the sum of squares of the three most severe injuries, regardless of body region injured. The TRISS comprises the RTS and ISS indexes as well as the trauma type (blunt or penetrating) and the patient age^8^.

**Figure 1:**
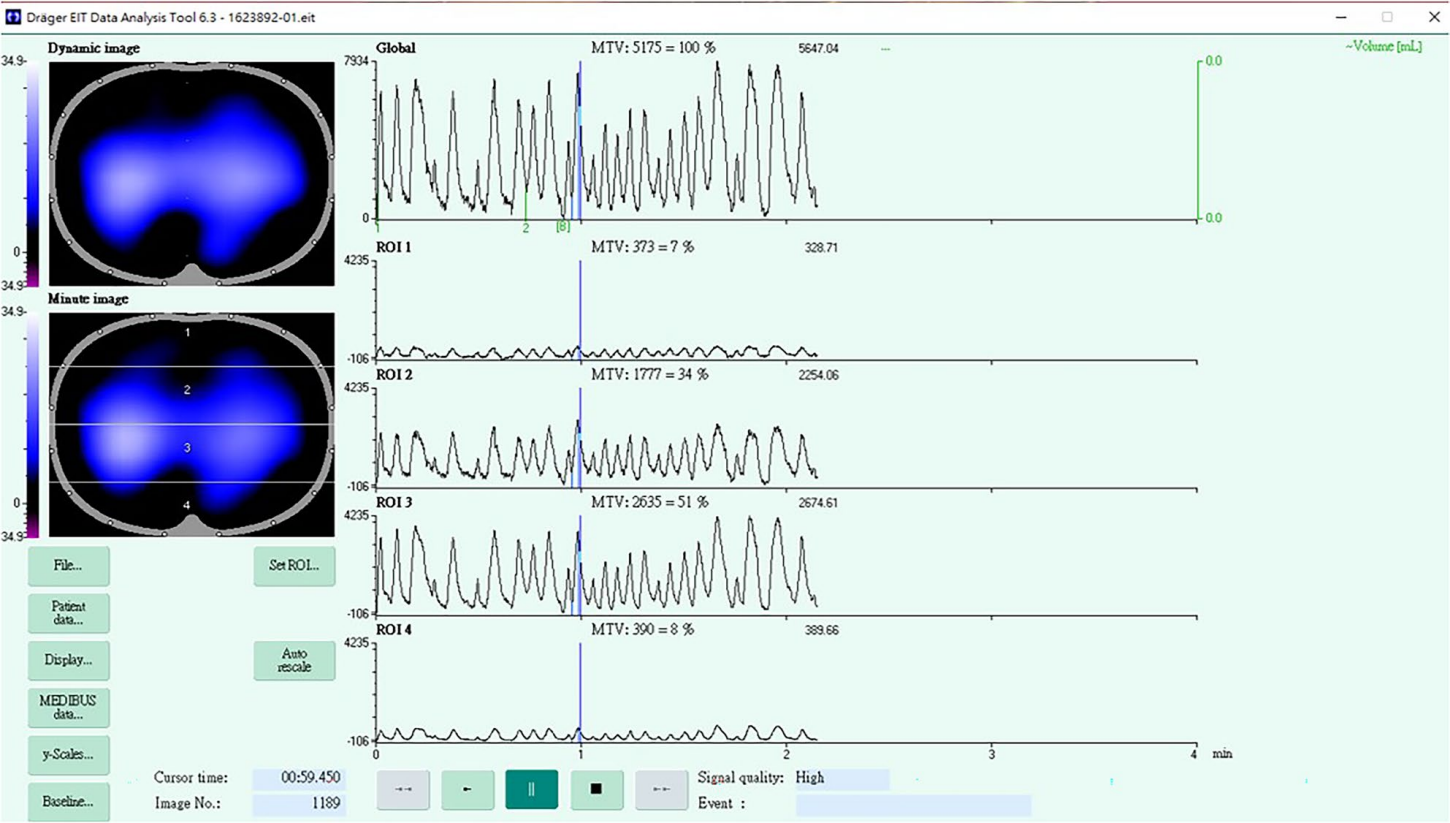
The presentation of EIT of the patient.

**Figure 2:**
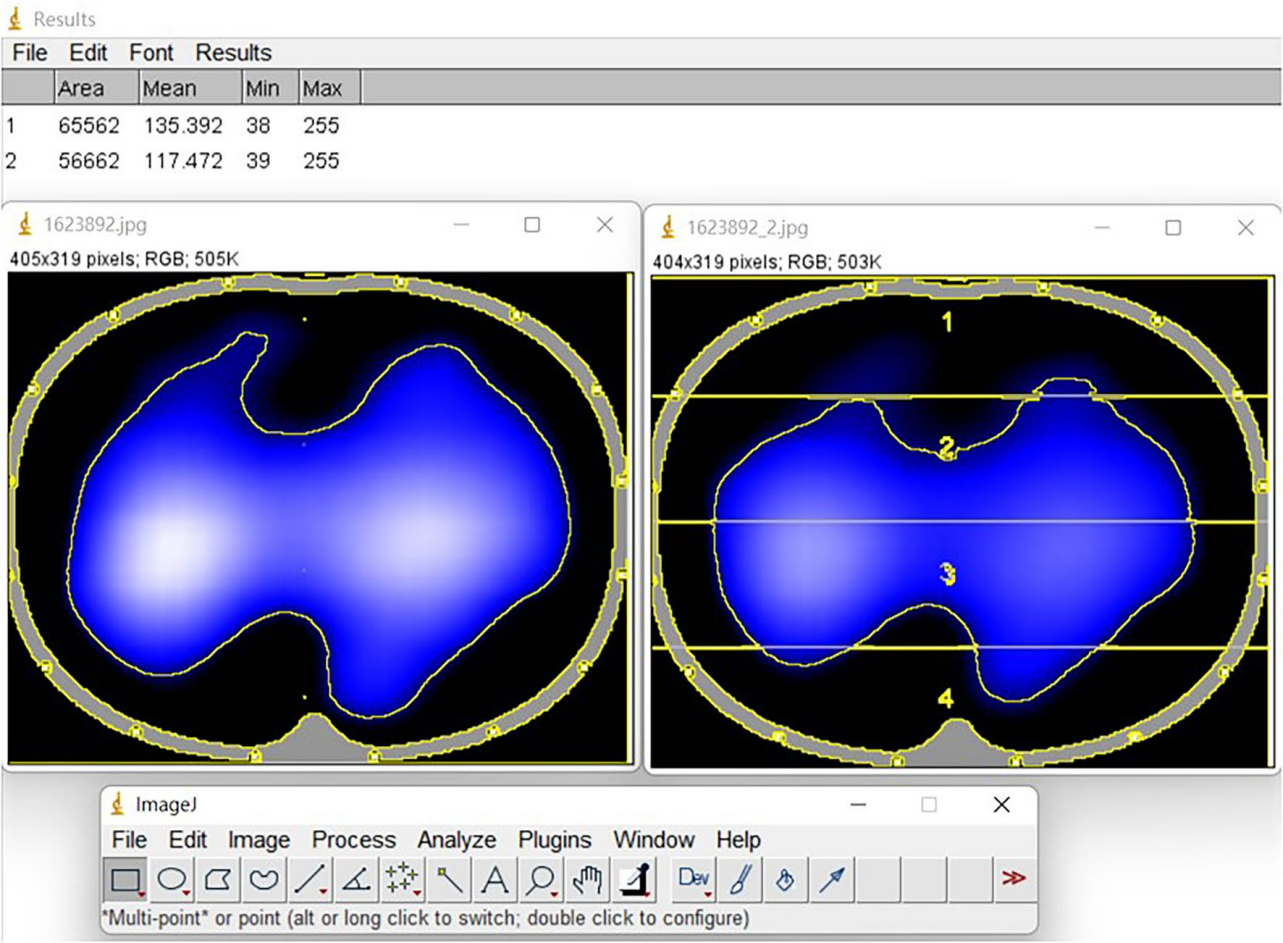
The method to measure the ventilated area at EIT images is shown in Image J.

Total patients with total severe chest trauma were divided into two groups: group that received and did not receive SSRF. The outcomes included hospital length of stay, ICU length of stay, and performance in the electrical impedance tomography (EIT) (Fig. 1). A group of sixteen X-ray transparent ECG electrodes (Blue Sensor BR-50-K, Medicotest, Ølstykke, Denmark) were positioned around the sixth intercostal space (medioclavicular line) on the thoracic circumference^9^. The EIT measurement technique, which involves detecting the potential variations that emerge from injecting tiny alternating electrical currents (50 kHz, 5 mA root mean square) through a series of surface electrodes in a rotating manner. It was always necessary to apply excitation currents between neighboring pairs of electrodes in order to measure voltage. All 16 drive electrode pairs received current injections during a single scanning cycle. Following each application of current to the body, potential changes between pairs of electrodes that did not carry current were measured^9^. Mechanical ventilation monitoring is the most promising use of ventilation EIT given that it gives real-time information on ventilation distribution at the bedside without requiring the patient and supported equipment to be transferred. Ventilation EIT imaging has been validated using established imaging techniques^9–11^. Delta ventilation of EIT and delta visual analog scale (VAS) before and after the surgery were also included. Delta ventilation of EIT image was calculated by Image J (Fig. 2).

A trauma team^12,13^ is a group of medical professionals who work together to provide immediate care to the severely injured patients. The team is typically composed of emergency physicians, trauma surgeons, nurses, respiratory therapists, and other specialists who are trained to handle life-threatening injuries. The primary goal of a trauma team is to stabilize the patient and prevent further injury or complications. The team works quickly and efficiently to assess the patient's condition, identify any life-threatening injuries, and provide the necessary treatment. Trauma teams are typically activated in response to severe injuries such as those resulting from motor vehicle accidents, falls, or other types of accidents that cause significant physical trauma in ER.

### Statistical analyses

In the statistical analysis of observational data, descriptive data are expressed as the mean ± standard deviation unless otherwise specified. Student’s t-test was used to an-alyze continuous variables, whereas the chi-squared test was used to compare categor-ical variables between patients who received SSRF and conservative treatment. The propensity score matching method was also used to match the patients of the two groups by age, sex, and different evaluation scale. Kaplan–Meier plots and log-rank tests were used to estimate the survival rate. A *p*-value of < 0.05 was considered to indicate statistical significance. Statistical analyses were performed using IBM SPSS Statistics version 20.0 (IBM Corp., Armonk, NY, USA). ImageJ was utilized in order to provide the graphical representation of the association between delta EIT and delta VAS (Fig. 2).

### Institutional review board statement

This study was approved by the Institutional Review Board/Ethics Committee of Tri Service General Hospital (1-108-05-165).

## Results

### Demographic characteristics of the enrolled patients

A total of 8396 patients with trauma were admitted to the Tri-Service General Hospital between January 2015 and June 2020. Among them, 1529 (18.21%) had major trauma with ISS of > 16 and admitted to the ICU initially. In addition, 596 patients had an AISthorax ≥ 3, and 519 (87%) survived. Table 1 summarizes the survival and expired groups according to sex, age, involvement or non-involvement of a trauma team, presence or absence of SSRF, and scores in the different injury evaluation methods. Except for sex, significant differences were noted in age, involvement or noninvolvement of a trauma team, presence or absence of SSRF, and scores in the different injury evaluation methods. Younger age (48.65 ± 19.28, *p* = 0.011), trauma team involvement (*p* < 0.001), presence of SSRF (*p* < 0.001) and lower injury severe scores (24.13 ± 7.17, *p* < 0.001) lead to a better survival rate. The trauma team was involved in 260 cases (43.6%), and 190 (73%) patients survived. Moreover, 74 (12.4%) patients received SSRF, and all of them survived.

**Table 1:** Demographic characteristics of major trauma cases with AIS_thorax_ ≥ 3.

	Survival (n = 519, 87%)	Expired (n = 77, 13%)	*p* value (0.05)
Gender			0.959
Male (432)	376 (72.45)	56 (72.73)	
Female (164)	143 (27.55)	21 (27.27)	
Age	48.65 ± 19.28	54.77 ± 22.15	0.011*^b^
Trauma team			< 0.001*^a^
Yes (260)	190 (36.61)	70 (90.9)	
No (336)	329 (63.39)	7 (9.09)	
SSRF			< 0.001*^a^
Yes (74)	74 (14.26)	0	
No (522)	445 (85.74)	77 (100)	
ISS	24.13 ± 7.17	33.82 ± 8.05	< 0.001*^b^
NISS	27.01 ± 7.92	36.68 ± 8.93	< 0.001*^b^
TRISS	0.88 ± 0.18	0.49 ± 0.32	< 0.001*^b^
RTS	7.25 ± 1.19	4.87 ± 2.44	< 0.001*^b^

*SSRF* surgical stabilization of rib fractures, *ISS* injury severity scale, *NISS* new injury severity score, *TRISS* trauma and injury severity score, *RTS* revised trauma score.

*Statistical significance was set at *p* < 0.05.

^a^Significance was assessed using χ^2^ tests. ^b^Significance was assessed using Student’s *t* tests.

### Demographic characteristics of specific chest trauma cases

To focus on chest trauma cases more specifically, patients with AISlimb > 3, AISabd > 3, AIShead > 3, and AISchest < 3 were excluded. Moreover, 249 (41.7%) patients in intensive care unit had major chest trauma, and their characteristics are summarized in Table 2, with the same parameters as Table 1. Except for sex, significant differences were found in age, involvement or noninvolvement of a trauma team, presence or absence of SSRF, and scores in the different injury evaluation methods. Younger age (49.17 ± 18.17, *p* = 0.025), trauma team involvement (*p* < 0.001), presence of SSRF (*p* = 0.027) and lower injury severe scores (21.62 ± 5.06, *p* < 0.001) led to better survival. A trauma team was involved in 77 (30%) patients, and 65 (84.4%) survived. Moreover, 62 (24.8%) patients received SSRF, and all of them survived.

**Table 2:** Demographic characteristics of specific chest trauma cases with AIS_thorax_ ≥ 3 in intensive care unit (patients with AISlimb > 3, AISabd > 3, AIShead > 3, and AISchest < 3 were excluded).

	Survival (n = 235, 94%)	Expired (n = 14, 6%)	*p* value
Gender			0.714
Male (185)	174 (74.66)	11 (65)	
Female (64)	61 (25.34)	3 (35)	
Age	49.17 ± 18.17	60.64 ± 23.99	0.025*^b^
Trauma team			< 0.001*^a^
Yes (77)	65 (31.16)	12 (90)	
No (172)	170 (68.84)	2 (10)	
SSRF			0.027*^a^
Yes (62)	62 (26.38)	0	
No (187)	173 (73.62)	14 (100)	
ISS	21.62 ± 5.06	30.07 ± 9.56	< 0.001*^b^
NISS	25.38 ± 6.78	32.86 ± 11.26	< 0.001*^b^
TRISS	0.92 ± 0.145	0.55 ± 0.338	< 0.001*^b^
RTS	7.51 ± 1.01	5.32 ± 2.879	< 0.001*^b^

*SSRF* surgical stabilization of rib fractures, *ISS* injury severity scale, *NISS* new injury severity score, *TRISS* trauma and injury severity score, *RTS* revised trauma score.

*Statistical significance was set at *p* < 0.05.

^a^Significance was assessed using χ^2^ tests. ^b^Significance was assessed using Student’s *t* tests.

### Further analysis of the prognostic factors for survival in patients with severe chest trauma

Possible prognostic factors for survival are listed in Table 3. The significant prognostic factors for survival rate in severe chest trauma cases were age < 65 years (HR = 17.727, *p* < 0.001) and trauma team involvement (HR = 9.953, *p* = 0.016). Among the scales used to evaluate the severity of trauma, the RTS is the most relevant prognostic factor (HR = 9.953, *p* = 0.005). At Table 3, we added SSRF in regression analysis. However, we used IBM SPSS Statistics for regression analysis of SSRF in survival status. Because of great disparity between two groups, the patients with SSRF were all survived and only 14 patients died without SSRF. The HR showed 130732070. This is a statistical bias. So, we take it out to further analysis. But, the close relationship between SSRF and survival was also improved at Table 2 (*p* = 0.027).

**Table 3:** Cox regression analysis for survival in severe chest trauma patients with AIS_thorax_ ≥ 3 (patients with AISlimb > 3, AISabd > 3, AIShead > 3, and AISchest < 3 were excluded).

Age (≥ 65 vs < 65)	Univariant analysis HR (95%, CI)		Multi-variant analysis HR (95%, CI)	
	17.727 (3.617–86.886)	< 0.001*	7.435 (2.379–23.239)	0.001*
Trauma team (Yes vs No)	9.953 (1.524–65.003)	0.016*	15.785 (3.439–72.456)	< 0.001*
SSRF (Yes vs No)	Unreadable^#^			
ISS	1.282 (0.99–1.66)	0.06		
NISS	0.875 (0.707–1.084)	0.222		
RTS	0.618 (0.443–0.862)	0.005*	0.571 (0.441–0.739)	< 0.001*

*SSRF* surgical stabilization of rib fractures, *ISS* injury severity scale, *NISS* new injury severity score, *RTS* revised trauma score.

^#^We used IBM SPSS Statistics for regression analysis of SSRF in survival status. Because of great disparity between two groups, the patients with SSRF were all survived and only 14 patients died without SSRF. The HR showed 130,732,070. This is a statistical bias.

*Statistical significance was set at *p* < 0.05.

### The outcomes of SSRF in patients with severe chest trauma

Of the 249 patients with severe chest trauma, 62 (24.9%) received SSRF. The two groups (surgical intervention and conservative treatment) were compared according to sex, age, hospital length of stay, ICU length of stay, Delta V of EIT, and ISS (Table 4). No significant differences were noted in sex (*p* = 0.307), age (*p* = 0.405), hospital length of stay (p = 0.792), and ISS (*p* = 0.985). The SSRF group had shortened ICU length of stay (5.24 ± 2.793 vs 8.27 ± 8.934, p = 0.045). Moreover, a statistically significant difference was noted in Delta V of EIT. The SSRF group had higher levels of Delta V in EIT (23.46 ± 7.89, p < 0.001).

**Table 4:** The outcomes of SSRF in severe chest trauma patients with AIS_thorax_ ≥ 3 (patients with AISlimb > 3, AISabd > 3, AIShead > 3, and AISchest < 3 were excluded).

	SSRF (n = 62, 25%)	Conservative (n = 187, 75%)	*p* value (0.05)
Gender			0.307
Male (183)	49 (79.03)	134 (71.7)	
Female (64)	13 (20.97)	53 (28.3)	
Age	51.50 ± 16.179	49.22 ± 19.419	0.405
Hospital length of stay (day)	18.31 ± 12.877	17.64 ± 18.302	0.792
ICU length of stay (day)	5.24 ± 2.793	8.27 ± 8.934	0.045*^b^
Delta V of EIT	23.46 ± 7.89	10.23 ± 8.37	< 0.001*^b^
ISS	21.94 ± 5.185	21.95 ± 5.745	0.985
NISS	26.82 ± 6.720	25.28 ± 7.333	0.144
TRISS	0.924 ± 0.136	0.897 ± 0.185	0.292
RTS	7.61 ± 1.068	7.35 ± 1.241	0.15

*SSRF* surgical stabilization of rib fractures, *EIT* Electrical impedance tomography, *ICU* intensive care unit, *ISS* injury severity scale, *NISS* new injury severity score, *TRISS* trauma and injury severity score, RTS, revised trauma score.

*Statistical significance was set at *p* < 0.05.

^a^Significance was assessed using χ^2^ tests.

^b^Significance was assessed using Student’s *t* tests.

## Discussion

### What is the difference between trauma scores?

Multiple scoring systems are available to evaluate and classify the severity of multiple injuries sustained by a patient. Among them, the ISS is the most common method of assessing injury patterns, prioritizing treatment, and predicting patient outcomes. The ISS, NISS, RTS, and TRISS are all trauma severity scores used to assess the severity of a patient's injuries and predict their outcomes^14^. According to the previous prospective observational study^8^, this was a study on two hundred elderly trauma patients over a consecutive period of 18 months. On the day of admission, data were collected from each patient to compute the ISS, NISS, RTS, and TRISS. Here, the TRISS was the strongest predictor of mortality in elderly trauma patients when compared to the ISS, NISS and RTS^8^. Dr. Mehmet Hilmi Hoke and colleagues included a total of 426 cases in their study^14^ and it revealed that TRISS was the most effective score for predicting mortality (area under the curve [AUC]: 0.93, sensitivity: 97.1%, and specificity: 76.8%). Although the TRISS has been widely utilized for mortality risk adjustment in trauma, its applicability in contemporary trauma populations is becoming increasingly contested. Dr. Georgios Filippatos presented a retrospective cohort study^15^ of admitted trauma patients conducted in two tertiary Greek hospitals from January 2016 to December 2018. There were 8,988 trauma patients in all, and 854 of them (9.5%) passed away. With an AUC of 0.912 (95% CI 0.902–0.923), the TRISS demonstrated outstanding discrimination; its results were equivalent to those of the TRISS (AUC = 0.908, 95% CI 0.897–0.919, *p* = 0.1195). The TRISS produced adequate calibration (Hosmer–Lemeshow test *p* = 0.113) and a statistically significant improvement in discrimination (AUC = 0.927, 95% CI 0.918–0.936, *p* < 0.0001).

In this retrospective study, trauma team involvement was found to result in better survival rates. According to Petrie et al.^16^, patients with an ISS of > 12 had a significantly better outcomes when a trauma team was activated than when they were treated on a service-by-service basis. A trauma team should be activated immediately in response to a critical or life-threatening injury or situation. The precise criteria for activating a trauma team may vary depending on the protocols and resources available in healthcare settings; however, generally, it is initiated when the patient’s condition is deemed severe and requires a multidisciplinary approach for rapid assessment, resuscitation and treatment. A well organized trauma team carries out complete resuscitation in a mean of 56 min versus 122 min, more than halving the total resuscitation time^17^. In our study, we also used ISS, NISS, TRISS, and RTS to evaluate the severity of trauma cases. Regardless of which method is used to assess the cases, higher scores indicate more severe traumatic situations and lead to higher mortality rate. Here, the RTS was found to be the most relevant prognostic factor (Table 3) for survival among the four different scales.

### The benefit of SSRF in chest trauma patients

The practice of SSRF for severe chest wall injury has exponentially increased over the last decade because of improved outcomes compared with nonoperative management. Several randomized controlled trials and multiple systematic reviews and meta-analysis have demonstrated the benefit of SSRF compared with nonoperative management in terms of pneumonia rate, duration of mechanical ventilation, hospital and ICU length of stay, and cost-effectiveness^18–22^. The benefit of SSRF is not only limited to flail chest cases. In this retrospective study, severe chest trauma cases benefit from SSRF with short ICU length of stay (5.24 ± 2.793 vs 8.27 ± 8.934, *p* = 0.045) and better performance in EIT examination (23.46 ± 7.89, *p* < 0.001). Although only 62 (26.38%) patients with severe chest trauma had received SSRF, all of them survived. In the long term, sustaining rib fractures has been associated with chronic pain, disability, and decreased quality of life^22–25^. To date, the practice of rib fixation or SSRF has increased exponentially and is now implemented in most international trauma centers^6,26,27^. The advantages of thoracoscopy or video-assisted thoracoscopic surgery was to help clearance of pleural cavity for better expansion of lung and prevention of empyema. Besides, precise localization of rib fractures by thoracoscopy also could decrease operation time and length of incision wounds^28^.

### Why did we use EIT for measure the outcome of SSRF?

Originally, we used pulmonary function tests or computed tomography of chest to evaluate lung function of chest trauma patients. Especially in severe chest trauma patients at ICU, it is difficult to get enough and effective information to evaluate the degree of improvement after treatments. Therefore, we used EIT in these severe chest trauma patients and investigate the safety and outcomes. The EIT uses electric current to evaluate the distribution of alternating current conductivity within the thoracic cavity. It is a non-invasive, bedside radiation-free functional imaging modality for continuous monitoring of lung ventilation and perfusion^29,30^. EIT offers several advantages over other imaging techniques, such as: (1) Non-invasiveness: EIT does not require the use of ionizing radiation or contrast agents, making it a safer alternative to other imaging techniques. (2) Real-time imaging: EIT can provide real-time images of the body's internal structures, making it useful in monitoring physiological processes such as lung function. (3) Portability: EIT equipment is smaller and less costly than other imaging equipment, making it more accessible and easier to use in a variety of settings. (4) High temporal resolution: EIT can provide images at a high temporal resolution, allowing for the monitoring of rapid physiological changes. The use of EIT in medical imaging is particularly useful in the monitoring of lung function. EIT can be used to monitor ventilation distribution and lung perfusion. EIT can also be used to adjust positive end-expiratory pressure (PEEP) settings in mechanical ventilation, which can help improve oxygenation and reduce ventilator-induced lung injury. Our patients who underwent SSRF showed higher delta V in EIT (Table 3). Delta V refers to the change in the volume between the inspiratory and respiratory periods, indicating the compliance of the thoracic cavity. Therefore, SSRF is significantly improving pulmonary ventilation functions for severe chest trauma.

### Limitations in research

Although we have enough chest trauma patients, the sample size of SSRF patients is still small. We used EIT in trauma patients in recent years, so we deleted many SSRF pateints with lack of available data. We believed more enrolled patients could make more credible results. Many previous studies utilized questionnaires to evaluate surgical outcomes. But this is hard to respond real situations with many biases. In this study, we have two nurse practitioners to perform EIT and record the data. We believed the difference of their technique errors was low. The hospital stay or ICU stay might be influenced and delayed by clinical practices or family support. This study is also a retrospective study in a single institute medical center. In the future, we will enroll more patients for statistical analysis despite of current meaningful results. This is a novel study about the utilization of EIT in trauma patients. There is lack of prior research studies on this topic. We hoped more and more studies of EIT in trauma patients in the future can support our conclusions.

## Conclusions

In patients with major thoracic injury, older age and higher revised trauma scales are associated with higher mortality rates. RTS is the significant score for evaluation of survival in severe chest trauma patients. The advantages of thoracoscopy or video-assisted thoracoscopic surgery was to help clearance of pleural cavity for better expansion of lung and prevention of empyema. Thoracoscopic assisted SSRF also reduced the ICU length of stay and helped patients to get better ventilation by EIT measurment. Therefore, thoracoscopic-assisted rib fixation with titanium plates and screws is feasible and suggested in severe chest trauma cases.

## Data Availability

The datasets analysed during the current study are not publicly available due to personal privacy but are available from the corresponding author on reasonable request.
